# A Scoping Review of Antimicrobial Therapy in *Leptospira* Infections in Domestic Animals

**DOI:** 10.3390/ani15203045

**Published:** 2025-10-20

**Authors:** Julia Mendes, Luiza Aymée, Walter Lilenbaum

**Affiliations:** Laboratory of Veterinary Bacteriology, Biomedical Institute of Fluminense Federal University, Niterói 24210-030, RJ, Brazil; ju_mendes@id.uff.br (J.M.); luizaaymeeps@gmail.com (L.A.)

**Keywords:** treatment, leptospirosis, livestock, dogs, horses, guidelines

## Abstract

**Simple Summary:**

Leptospirosis is a worldwide zoonosis that affects both companion and production animals, causing severe health and economic impacts. This scoping review systematically mapped and critically analyzed antimicrobial treatment protocols across species. The findings revealed high heterogeneity in drug dosages. Importantly, most studies lacked standardized protocols, adequate monitoring of bacterial clearance, and recent clinical trials. By synthesizing dispersed evidence, this review highlights areas of consensus, identifies critical gaps, and underscores the urgent need for harmonized, evidence-based guidelines to improve animal health, reduce economic losses, and mitigate public health risks within a One Health perspective.

**Abstract:**

Leptospirosis, caused by *Leptospira* spp., affects multiple domestic species and can result in significant economic and public health impacts. This scoping review, conducted following the PRISMA 2020 guidelines, searched PubMed, SciELO, and Scopus for original studies that described complete therapeutic protocols (including dose, duration, and confirmed diagnosis) in dogs, cats, cattle, pigs, horses, sheep, and goats. Thirty-five studies met the criteria: 14 (40%) in cattle, 7 (20%) in swine, 2 (5.7%) in small ruminants, 7 (20%) in dogs and cats, and 5 (14.3%) in horses. In livestock, streptomycin monotherapy has predominated, demonstrating high efficacy against renal and genital carriers, but it faces regulatory restrictions in several countries. In companion animals, treatment often addressed acute cases using doxycycline and aminopenicillins, with frequent drug combinations. Horses were mainly treated with penicillin, alone or associated with other agents. Across species, protocols showed substantial heterogeneity, lack of harmonization, and limited evaluation of bacteriological cure, with most studies published before 2000. This scarcity of recent clinical trials reinforces the neglected status of animal leptospirosis. By compiling fragmented evidence, this review identifies converging practices that may serve as a preliminary consensus, highlights discrepancies and knowledge gaps, and provides an evidence-based framework to support the development of standardized, species-specific guidelines urgently needed in a One Health context.

## 1. Introduction

Leptospirosis is an infectious and zoonotic disease that is widely distributed globally [1]. The etiological agent is the bacterium *Leptospira* spp., characterized by a thin, long, and helical morphology with a single well-developed flagellum, enabling high mobility through spiral movements [2]. The disease can occur in several animal species, including domestic ones, resulting in significant economic and health impacts [3,4]. Leptospirosis in animals is caused mainly by the species *Leptospira interrogans*, *L. borgpetersenii*, and *L. kirschneri*, which encompass several pathogenic serovars classified as incidental or adapted strains [5,6]. Host-adapted strains are those maintained by a specific maintenance host, without requiring other species to sustain their transmission cycle. These strains typically cause chronic, asymptomatic, or subclinical infections in the maintenance host. However, when they infect incidental or accidental host species to which they are not adapted, they may lead to acute clinical disease. In such cases, these strains are referred to as incidental or non-adapted strains [7].

The disease is more prevalent in tropical regions, which are characterized by high temperatures and heavy rainfall [8]. Transmission occurs primarily through exposure to water or soil contaminated with the urine, genital fluids, or tissues of infected animals. It can also occur through direct contact between animals [9]. In urban areas, the disease manifests itself in acute outbreaks, especially in areas with low socioeconomic development, where the lack of basic sanitation favors the proliferation of rodents and human and animal contact (mainly dogs) with the bacteria present in contaminated urine [1,9]. This scenario is aggravated during periods of heavy rainfall and floods. The socioeconomic impacts are significant: it overloads health systems due to the high demand for hospitalizations, reduces labor productivity due to absences, and, in severe cases, increases mortality in vulnerable populations [8]. Furthermore, the costs of health surveillance and rodent control represent a financial challenge for governments and affected communities [10]. Acute cases in humans and dogs are often associated with strains such as those of Icterohaemorrhagiae serogroup; they lead to severe cases of multiple-organ failure, jaundice, hematuria, fever, and renal failure, and can be fatal [1,11].

In rural areas, leptospirosis presents itself in both acute and chronic forms. The persistence of the bacteria in stagnant water, moist soils, and contaminated environments facilitates transmission between production, companion, and wild animals, perpetuating the disease cycle [12]. The economic consequences are severe, including losses in milk and meat production, outbreaks of abortions in herds, declines in animal fertility, and high veterinary treatment costs [4]. These factors compromise the income of small producers, exacerbate rural inequalities, and threaten food security. Chronic infections, usually linked to adapted strains such as those of serogroup Sejroe in cattle, often go unnoticed but cause significant reproductive losses due to subfertility, embryo mortality, and abortions [13,14].

A protocol for leptospirosis prevention and control was suggested for bovines, based on three key pillars: environmental management, vaccination, and treatment of carriers [15]. Although originally designed for cattle, this triad can be extrapolated to the control of leptospirosis in other animal species.

Among these, the greatest challenge is intervening in the environmental component of the epidemiological cycle. It can be difficult in tropical regions and for animals maintained under extensive production systems. In contrast, for species kept in more controlled environments, such as intensive commercial swine production, control is more feasible. Still, environmental management is preventive, and it must be associated with the other two pillars for effective control [16].

Vaccination is the most economically viable strategy for livestock, as it can be applied to the entire herd [17]. Besides livestock, it is also commonly used in dogs and horses. Although the effectiveness of leptospiral vaccines in inducing humoral and cellular immune responses depends on both the animal species and the antigenic composition of the vaccine, immunization is known to reduce the severity of clinical signs in infected animals. However, the ability of leptospiral vaccination in preventing renal or genital colonization and eliminating the carrier state is controversial. Additionally, some commercial vaccines may lack strains representing serogroups that are epidemiologically relevant in specific regions.

The treatment of leptospiral carriers with antimicrobials remains the only effective strategy to stop colonization and interrupt bacterial shedding [3]. However, this approach relies on the prior identification of carrier animals through highly sensitive and specific diagnostic tools, particularly molecular methods such as Polymerase Chain Reaction (PCR), which increases the overall costs of this approach. A major limitation is regarding the use of antimicrobials themselves, as it may contribute to the emergence of antimicrobial resistance, either within the commensal microbiota or potentially in leptospires, although the latter remains underexplored.

Several antimicrobial classes have been described as effective against *Leptospira* spp., such as aminoglycosides, tetracyclines, β-Lactams, and fluoroquinolones [16,18,19]. These agents are classified as either bactericidal or bacteriostatic according to their mechanism of action. Bactericidal agents are capable of killing bacteria, whereas bacteriostatic agents inhibit bacterial growth without directly causing cell death [20]. Among the antibiotics commonly used in the treatment of leptospirosis in animals, five groups deserve to be highlighted: aminoglycosides, tetracyclines, β-lactams, macrolides, and fluoroquinolones.

The first of them, aminoglycosides, have bactericidal effects and act on bacterial ribosomes, inhibiting protein synthesis [21]. The most widely used member of this class is streptomycin, but gentamicin has also shown good action against *Leptospira* strains in vitro [22]. Tetracyclines also inhibit bacterial protein synthesis, but are bacteriostatic. In this group, oxytetracycline is the most commonly used and well-documented agent for treating leptospirosis in livestock [23,24], while doxycycline has been widely used to treat leptospirosis in dogs [16]. The β-Lactams are bactericides and act by inhibiting the synthesis of the bacterial cell wall. Natural penicillins, aminopenicillins (ampicillin and amoxicillin), and second- and third-generation Cephalosporins (cefoxitin, ceftriaxone, and cefotaxime) have been used to treat leptospiral infections [25]. Finally, fluoroquinolones are also bactericidal agents that inhibit the replication of bacterial DNA; the most commonly used drug in this class is enrofloxacin, which has been used mainly in dogs, cats, and pigs [16,26].

Given the importance of appropriate antimicrobial treatment for leptospiral control, as well as the wide range of antimicrobials employed and the lack of standardized protocols across animal species, this scoping review aimed to systematically map and critically analyze the available evidence on antimicrobial therapy for leptospirosis in domestic animals. In addition to describing therapeutic practices, the study sought to identify areas of consensus, highlight inconsistencies across species, and expose persistent critical gaps. By synthesizing disparate and heterogeneous data, the review aims to provide a structured basis that supports the harmonization of national and international treatment guidelines and determine bacteriological diagnostic methods of cure, serving as a reference for both practitioners and policymakers.

## 2. Materials and Methods

A comprehensive search was first conducted in major databases and in the International Prospective Register of Systematic Reviews (PROSPERO) to identify existing systematic reviews or meta-analyses on the topic. No such studies were found. Therefore, a literature review was undertaken, following the updated 2020 guidelines of the Preferred Reporting Items for Systematic Reviews and Meta-Analyses (PRISMA) [27].

The literature search was carried out in PubMed https://pubmed.ncbi.nlm.nih.gov (accessed on 30 April 2025), SciELO https://search.scielo.org (accessed on 30 April 2025), and Scopus https://www.scopus.com (accessed on 30 April 2025), with no year restrictions, and included studies published up to April 2025. The same search terms were applied across all databases: (leptospir * OR leptospirosis) AND (treatment OR therapy OR antibiotic OR antimicrobial OR “antibiotic treatment”) AND (pigs OR swine) AND (horses OR equines) AND (sheep OR goats OR ruminants) AND (cattle OR bovine OR ruminants OR cows OR herds OR bulls) AND (dogs OR cats).

The search followed a two-stage screening process: the first involved title and abstract screening, and the second consisted of full-text review by two independent reviewers. A predefined data extraction spreadsheet was used and filled out by each reviewer.

Inclusion criteria encompassed original studies (observational studies, clinical trials, case reports, case series, or controlled trials) with a clinical-therapeutic focus, published in peer-reviewed journals. Eligible studies were expected to describe a complete antimicrobial treatment protocol for leptospirosis specifying drug dosages, duration of therapy, and methods for confirming diagnosis in dogs, cats, cattle, pigs, horses, goats, or sheep, published up to April 2025.

Exclusion criteria included conference abstracts, letters to the editor, studies conducted exclusively in humans, studies lacking a therapeutic focus or presenting inconsistent or incomplete methodologies, those without a confirmed diagnosis by at least one recommended method, or clinical evaluations where no outcome was reported due to death or loss to follow-up.

The results were categorized into three main groups listing the type of therapy and the most commonly used antimicrobials: (1) livestock-comprising sheep, goats, pigs, and cattle, focusing on asymptomatic individuals or those presenting reproductive disorders, treated predominantly with monotherapy; (2) companion animals—including dogs and cats, emphasizing the clinical treatment of systemic symptoms through the use of two or more antimicrobials; and (3) working animals—specifically horses presenting systemic and reproductive manifestations, treated using sequential monotherapy or combination antimicrobial protocols.

## 3. Results

**Figure 1:**
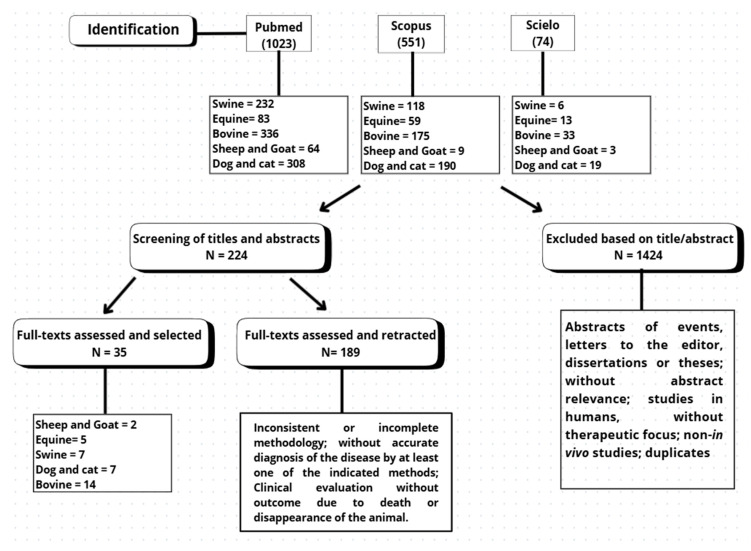
Flow of articles through the phases of the systematic review.

**Figure 2:**
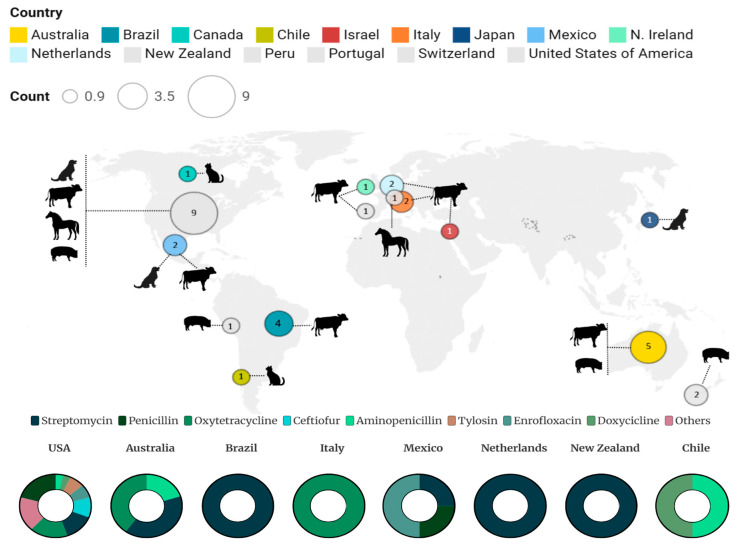
Distribution of animals and antimicrobial treatments across countries in the selected studies.

Our search retrieved 1023 studies in PubMed, 551 studies in Scopus, and 74 studies in SciELO. The distribution of studies by species is represented in Figure 1. After a careful screening of their abstracts, 224 studies met the initial criteria. Following a full-text review and the application of inclusion and exclusion criteria, 35 studies were retained for detailed analysis (Figure 1). The geographic distribution, as well as the studied animal species, is demonstrated in Figure 2.

Based on this, we selected for a detailed analysis two studies on small ruminants (sheep and goats), five on horses, seven studies on pigs, seven on dogs and cats, and 14 on cattle. No chronological limitations were applied to the search, which resulted in 29 (70.7%) of the results being more than 10 years ago. Of these, 17 (58.6%) were published before 2000.

### 3.1. Antimicrobial Therapies for the Treatment of Leptospirosis in Livestock

The treatment of livestock (cattle, small ruminants, and swine) was investigated in 23 studies, which were categorized into two distinct scenarios: (a) asymptomatic animals identified as leptospiral carriers, characterized as maintenance hosts, naturally or experimentally infected, treated to interrupt the epidemiological cycle, that is, to interrupt the *Leptospira* shedding, to reduce environmental dissemination and the risk of transmission to other animals and humans; (b) animals that presented clinical signs of leptospirosis, with mostly chronic and reproductive manifestations, treated to resolve acute illness and achieve clinical recovery (Table 1).

Among the 23 studies, 15 (65.2%) involved asymptomatic animals. These included nine studies in cattle (9/14, 64.3%), one in small ruminants (1/2, 50%), and five in swine (5/7, 71.4%). Of these 15 studies, ten addressed experimentally infected animals: six in cattle [18,28,29,30,31,32], one in sheep [33], and three in pigs [23,34,35]. The remaining five studies assessed naturally infected asymptomatic animals, three in cattle [36,37,38], and two in swine [39,40].

**Table 1:** Description of studies and therapeutic protocols for leptospirosis in livestock.

Reference	Species	Clinical	N	Antimicrobial	Dosage	Duration of Treatment	Therapeutic Outcome	Revaluation (Period/Test)
[34]	Swine	Asymptomatic (Experimental infection)	2	Oxytetracycline	1.8 g of feed/PO	15 days	Temporary suppression of leptospiruria	Once a week for 14 weeks/MAT, darkfield microscopy of urine
					1.4 g of feed/PO			
[39]	Swine	Asymptomatic (Natural infection)	111	Streptomycin	25 mg/kg/IM	Single dose	Described as effective therapy in all animals	One month and one year after treatment/MAT. **Leptospiral shedding not evaluated.**
[40]	Swine	Asymptomatic (Natural infection)	21	Streptomycin	25 mg/kg/IM	Single dose	Described as effective therapy in all animals. **Interruption of leptospiruria.**	12 to 45 days after treatment/MAT, darkfield microscopy of urine and culture
[41]	Swine	Abortions (Natural infection)	110	Streptomycin	25 mg/kg/IM	Three doses	Described as ineffective therapy due to the lack of clinical improval	Eight weeks after treatment/MAT, urine culture, clinical evaluation.
				Oxytetracycline	10 kg/ton of feed/VO	Four weeks		
[35]	Swine	Asymptomatic (Experimental infection)	8	Oxytetracycline	20 mg/kg/IM	Single dose	Temporary supression of leptospiruria. Described as ineffective since six of eight (75%) animals were positive in kidneys in the end of the study	For three months after treatment/MAT, and urine and kidney culture
[42]	Swine	Abortions, stillbirths, weak piglets, and mummified fetuses. (Natural infection)	23	Streptomycin	25 mg/kg/IM	Three doses	Described as effective therapy due to clinical improval	NA/Clinical evaluation. **Leptospiral shedding not evaluated.**
[23]	Swine	Asymptomatic (Experimental infection)	3	Streptomycin and penicilin	25 mg/kg/IM	Single dose	Described as effective therapy in all animals. **Interruption of leptospiruria.**	Seven to ten days after treatment/Urine culture, Fluorescent Antibody Test and histology
			2			Three doses		
			2			Five doses		
			4	Ceftiofur	5 mg/kg/IM	Three doses	Described as ineffective therapy. All animals remained with leptospiruria.	
			4		20 mg/kg/IM	Three doses		
			4		5 mg/kg/IM	Five doses		
			4		20 mg/kg/IM	Five doses	Described as effective in 1/4 (25%) of animals.	
			4	Oxytetracycline	10 mg/kg/IM	Three doses		
			4		40 mg/kg/IM	Three doses	Described as effective therapy in all animals. **Interruption of leptospiruria.**	
			4		10 mg/kg/IM	Five doses	Described as effective in 3/4 (75%) of animals	
			4		40 mg/kg/IM	Five doses	Described as effective in 3/4 (75%) of animalsarb	
			4	Erythromycin	4 mg/kg/IM	Three doses	Described as ineffective therapy. All animals remained with leptospiruria.	
			4		25 mg/kg/IM	Three doses	Described as effective in 3/4 (75%) of animals	
			4		4 mg/kg/IM	Five doses		
			4		25 mg/kg/IM	Five doses		
			4	Tylosin	8.8 mg/kg/IM	Three doses		
			4		44 mg/kg/IM	Three doses		
			4		8.8 mg/kg/IM	Five doses		
			4		44 mg/kg/IM	Five doses	Described as effective therapy in all animals. **Interruption of leptospiruria.**	
			4	Ampicillin	10 mg/kg/IM	Three doses	Described as ineffective therapy. All animals remained with leptospiruria.	
			4		50 mg/kg/IM	Three doses		
			4		10 mg/kg/IM	Five doses		
			4		50 mg/kg/IM	Five doses		
			4	Tiamulin	5 mg/kg/IM	Three doses		
			4		25 mg/kg/IM	Three doses	Described as effective therapy in all animals. **Interruption of leptospiruria.**	
			4		5 mg/kg/IM	Five doses	Described as ineffective therapy. All animals remained with leptospiruria.	
			3		25 mg/kg/IM	Five doses	Described as effective in 1/3 (33.3%) of animals.	
[28]	Bovine	Asymptomatic (Experimental infection)	7	Streptomycin	25 mg/kg/IM	Single dose	Described as effective therapy in all animals.	For 45 days after treatment/MAT. **Leptospiral shedding not evaluated.**
[29]	Bovine	Asymptomatic (Experimental infection)	5	Streptomycin	25 mg/kg/IM	Single dose	Described as effective in 2/5 (40%) of animals.	Seven days after treatment/Culture of reproductive and kidney tissues
			5			Two doses	Described as effective in 1/5 (20%) of animals.	
[30]	Bovine	Asymptomatic (Experimental infection)	3	Streptomycin	25 mg/kg/IM	Single dose	Described as effective therapy in all animals. **Interruption of leptospiruria.**	For three weeks after treatment/Urine PCR
			3			Five doses		
[36]	Bovine	Asymptomatic (Natural infection)	3	Streptomycin	25 mg/kg/IM	Single dose	Described as effective therapy in all animals. **Interruption of leptospiruria.**	Four days after treatment/Urine PCR
[31]	Bovine	Asymptomatic (Experimental infection)	6	Amoxicillin	15 mg/kg/IM	Two doses	Described as effective therapy in all animals. **Interruption of leptospiruria.**	For seven weeks after treatment/Urine culture and Fluorescence Antibody Test
[43]	Bovine (Calf)	Systemic signs—Jaundice and hemoglobinuria (Natural infection)	NA	Oxytetracycline, Streptomycin and Chlortetracycline	Oxytetracycline 10 mg/kg/IM; Streptomycin 20 mg/kg/IM; Chlortetracycline 50 mg/kg/VO	Oxytetracycline Single dose, Streptomycin three doses; Chlortetracycline 12 days	Described as effective therapy due to clinical improval.	Seven days after treatment/Clinical evaluation. **Leptospiral shedding not evaluated.**
[18]	Bovine	Asymptomatic (Experimental infection)	3	Oxytetracycline	20 mg/kg/IM	Single dose	Described as effective therapy in all animals. **Interruption of leptospiruria.**	For four weeks after treatment/Urine culture, PCR, and antibody fluorescence test
			3		11 mg/kg/IM	Three doses	Described as effective in 2/3 (66.6%) of animals. **Interruption of leptospiruria.**	
			3	Tilmicosin	10 mg/kg/SC	Single dose	Described as effective therapy in all animals. **Interruption of leptospiruria**.	
			3	Streptomycin and Penicillin	25 mg/kg/IM	Single dose		
			3	Ceftiofur	20 mg/kg/IM	Three doses		
			3		2.2 mg/kg/IM	Three doses	Described as effective in 1/3 (33.3%) of animals.	
			3		2.2 mg/kg/IM	Five doses	Described as effective therapy in all animals. **Interruption of leptospiruria.**	
			3		5 mg/kg/IM	Three doses	Described as effective in 2/3 (66.6%) of animals	
			3		5 mg/kg/IM	Five doses	Described as effective therapy in all animals. **Interruption of leptospiruria**.	
			3	Tylosin	18 mg/kg/IM	Five doses	Described as effective in 2/3 (66.6%) of animals	
[32]	Bovine	Asymptomatic (Experimental infection)	9	Tulathromycin	2.5 mg kg/SC	Single dose	Described as effective in 8/9 (88.9%) of animals. **Interruption of leptospiruria**.	28 days after treatment/Urine and kidney culture and PCR
			10	Ceftiofur	6.6 mg/kg/SC	Single dose	Described as effective in 19/10 (90%) of animals	
[44]	Goats	Abortions (Natural infection)	48	Streptomycin	25 mg/kg/IM	Single dose	Described as was effective in 37/48 animals (89.6%). **Interruption of leptospiruria**.	One year after treatment/Urine PCR
[37]	Bovine	Asymptomatic (Natural infection)	5	Oxytetracycline	3 mg/kg/IM	Five doses	Described as effective therapy in all animals. **Interruption of leptospiruria.**	27 weeks after treatment/Urine PCR
[45]	Bovine	Reproductive failures (Natural infection)	24	Streptomycin	25 mg/kg/IM	Single dose	Described as effective therapy due to clinical improval.	One year after treatment/Clinical and reproductive evaluation. Leptospiral shedding not evaluated.
[38]	Bovine	Asymptomatic animals and animals with reproductive failures (natural infection)	98	Oxytetracycline	10 mg/kg/IM	Four doses	Described as effective therapy due to clinical improvement and reduced serological titers.	Five weeks after treatment/MAT. **Leptospiral shedding not evaluated.**
[46]	Bovine	Reproductive failures (Natural infection)	74	Streptomycin and Penicillin	Streptomycin (25 mg/kg) and Penicillin (15,000 UI/kg)/IM	Five doses	Described as effective in 53/74 (71.3%) of animals. **Interruption of leptospiruria.**	Five and 28 days after treatment/Urine PCR
			75	Enrofloxacin	15 mg/kg/IM	Five doses	Described as effective in 70/75 (93.4%) of animals. **Interruption of leptospiruria.**	
[33]	Sheep	Asymptomatic (Experimental infection)	9	Streptomycin	25 mg/kg/IM	Single dose	Therapy effective in only 1/9 (11.1%) of animals	Three and 35 days after treatment/Genital samples PCR. Leptospiruria not evaluated.
			8			Three doses	Described as effective therapy in all animals.	
[47]	Bovine	Reproductive failures (Natural infection)	13	Streptomycin	25 mg/kg/IM	Single dose	Described as effective in 7/13 (53.8%) of animals	Seven days after treatment/Genital samples PCR. Leptospiruria not evaluated.
			17			Three doses	Described as effective in 16/17 (94.1%) of animals	
[24]	Bovine (Calf)	Systemic signs—Jaundice and hemoglobinuria (Natural infection)	30	Oxytetracycline	20 mg/kg/IM	Single dose	Described as effective therapy due to clinical improval.	NA/Clinical evaluation

Eight studies from the selected ones in livestock focused on naturally infected animals exhibiting clinical manifestations of leptospirosis. Among them, five were conducted in cattle: three involving animals with reproductive disorders such as abortions, chronic infertility, and subfertility [45,46,47], and two involving calves with acute systemic signs, including hematuria and jaundice [24,43]. Notably, in the study by Grippi et al. [38], both clinically affected animals and asymptomatic animals in contact with them were treated. As for other livestock species, one study was conducted in goats with abortion cases from a single herd [44], and two studies involved sows with reproductive losses, including abortions and stillbirths [41,42].

The studies employed treatment protocols involving drugs from various antimicrobial classes, including aminoglycosides, tetracyclines, β-lactams, macrolides, and fluoroquinolones [16,18,19]. However, these protocols differed in terms of the number of drugs administered. Monotherapy, defined as the use of a single antimicrobial agent, was the most commonly adopted approach, reported in 12 out of 14 studies in cattle, both studies in small ruminants (2/2), and six studies in swine. In contrast, combined therapy, i.e., the use of two or more antimicrobial agents, was described in only two studies in cattle and one in swine.

#### 3.1.1. Streptomycin

Streptomycin was the most frequently used antimicrobial, as demonstrated in 16 of 23 livestock (69.6%): nine in cattle, two in small ruminants, and five in swine. This drug was used in monotherapy (12 of 16 studies, 75%) and associated with penicillin (3 of 16 studies, 18.7%) or oxytetracycline (1 of 16 studies, 6.3%). Treatment protocols involving streptomycin achieved bacteriological cure at a dosage of 25 mg/kg. This dosage was administered in different regimens according to the type of carrier state: a single dose for renal carriers [28,29,30,33,36,39,40,44,45,47], three doses for genital carriers [33,42,47], and five consecutive applications in some cases [30].

#### 3.1.2. Oxytetracycline

Oxytetracycline was the second most frequently used antimicrobial, reported in nine of the 23 studies (39.1%). Among these, five studies were conducted in cattle: four using oxytetracycline as monotherapy and one in combination with streptomycin. The remaining four studies were conducted in swine, all employing monotherapy. Long-acting formulations were used in two studies [24,35].

Dosage and treatment duration varied across studies. In cattle, reported dosages ranged from 3 mg/kg for five consecutive days [37] to a single 20 mg/kg dose [18,24]. In swine, dosages ranged from 10 to 40 mg/kg, administered in three to five doses [23,35]. Additionally, oxytetracycline was also administered via feed in some studies [34,41].

The most effective protocol in cattle was a single 20 mg/kg dose, which resulted in 100% efficacy [18,24]. In swine, this same dosage achieved 87.5% efficacy [35]. Another highly effective regimen in cattle was the 5-day treatment with 3 mg/kg, which also resulted in complete efficacy. In swine, experimental protocols comparing 10 and 40 mg/kg over three to five administrations showed that the 40 mg/kg dosage was the most effective [23].

#### 3.1.3. Ceftiofur

The third most frequently used antimicrobial was ceftiofur, a third-generation cephalosporin. It was employed in two cattle studies and one swine study, across a total of six treatment protocols. In swine, doses of 5 mg/kg and 20 mg/kg administered over three or five days were ineffective at eliminating the infection [23]. In contrast, the same regimens in cattle—5 mg/kg for five days and 20 mg/kg for three days—demonstrated complete efficacy. Additional protocols in cattle included a 2.2 mg/kg dose for five days [18] and a single 6.6 mg/kg subcutaneous injection [32], which showed 100% and 90% effectiveness, respectively.

#### 3.1.4. Macrolides

Macrolides were also evaluated in cattle and pigs via intramuscular administration. In cattle, tylosin at 18 mg/kg for five days achieved only 66.7% efficacy [18]. However, tilmicosin given as a single subcutaneous dose of 10 mg/kg resulted in 100% efficacy [18], while tulathromycin at 2.5 mg/kg provided 88.9% efficacy [32]. In swine, tylosin was tested at 8.8 mg/kg and 44 mg/kg over three- or five-day courses, with only the 44 mg/kg regimen proving effective (75% efficacy over three days and 100% over five days). Erythromycin was also tested in swine at doses of 4 mg/kg and 25 mg/kg for three and five days, respectively; only the five-day course at 25 mg/kg achieved 100% success [18].

#### 3.1.5. Other Drugs Used in Monotherapy

Aminopenicillins, such as amoxicillin and ampicillin, were used in cattle and swine, respectively. In cattle, amoxicillin administered at 15 mg/kg in two doses 48 h apart successfully treated all animals [31]. In contrast, four ampicillin protocols were tested in swine—10 mg/kg and 50 mg/kg administered for three or five days—but none of them proved effective in eliminating the infection [23].

A more recent study in cattle evaluated enrofloxacin, using five intramuscular doses of 15 mg/kg in a cohort of 75 animals, resulting in a 93.3% cure rate [46]. Tiamulin was tested in swine at doses of 5 mg/kg and 25 mg/kg for three or five days. Only the 25 mg/kg dose administered over three days yielded satisfactory efficacy [23].

#### 3.1.6. Combined Therapy in Livestock

Streptomycin has been used in combination with either oxytetracycline or penicillin. The combination with oxytetracycline was tested in swine and cattle. In swine, the protocol involved intramuscular streptomycin at 25 mg/kg for three days, combined with oxytetracycline administered in feed over four weeks. However, this regimen was deemed ineffective, as abortions continued to occur in the herd [41]. In cattle, combinations of streptomycin with either tetracyclines or penicillin were evaluated. Yeruham et al. [43] administered streptomycin (25 mg/kg IM for three days), a single dose of long-acting oxytetracycline (10 mg/kg IM), and oral chlortetracycline (50 mg/kg for 12 days), reporting clinical recovery.

The combination of streptomycin and penicillin was assessed in three studies. In one study, a single dose of 25 mg/kg of each antibiotic was effective in treating all three treated cattle [18]. Another study used streptomycin (25 mg/kg) combined with penicillin (15,000 IU/kg) for five consecutive days, but achieved success in only 21 of 74 animals (28.4%) [46]. In swine, the combination of streptomycin and penicillin at 25 mg/kg each, administered in one, three, or five doses, was effective in all cases [23].

### 3.2. Antimicrobial Treatment of Leptospirosis in Dogs and Cats

Studies on antimicrobial treatment of leptospiral infections in dogs and cats have primarily focused on animals presenting clinical signs of the disease. A total of seven studies were reviewed: one involving experimentally infected dogs [48], and six involving naturally infected animals (four in dogs and two in cats). The description of the studies is demonstrated in Table 2.

The animals presented with acute leptospirosis, with clinical signs including anorexia, lethargy, polyuria, polydipsia, hematuria, and azotemia. However, the clinical manifestations in cats were reported as mild, with fewer clinical signs than those reported in studies about dogs.

**Table 2:** Description of studies and therapeutic protocols for leptospirosis in dogs ans cats.

Reference	Species	Clinical	N	Antimicrobial	Dose	Duration of Treatment	Therapeutic Outcome	Revaluation (Period/Test)
[48]	Dog	Jaundice, hematuria, fever, anorexia and macroscopic changes in the kidneys and liver	18	Penicillin	50,000 UI/lb IM	Five days	Described as effective in 15/18 (83.3%) of animals due to clinical improval	Three weeks after treatment/Culture and Clinical evaluation. **Leptospiral shedding not evaluated.**
			13	Streptomycin	40 mg/kg IM	BID/Five days	Described as effective in 12/13 (92.3%) of animals due to clinical improval	
			10	Oxytetracycline	25 mg/lb PO	BID/Five days	Described as effective in 8/10 (80%) of animals due to clinical improval	
			10	Chlortetracycline	25 mg/lb PO	BID/Five days		
[49]	Dog	Fever, jaundice, loss of appetite, dehydration and vomiting	1	Penicillin	NA /IV	Four days	Described as ineffective due to the lack of clinical improval	13 days after starting treatment/Darkfield microscopy of urine and hematological evaluation
				Benzylpenicillin	10 mg/kg IV	BID/Five days		
				Doxycycline	10 mg/kg PO	BID/three weeka		
				Streptomycin	15 mg/kg IM	BID/Three days	Described as effective therapy due to clinical improvement	At the end of treatment (day 3)/Darkfield microscopy of urine
[50]	Cat	Polyuria, polydipsia, anorexia, lethargy, fever and dehydration, polyuria, polydipsia, and hematuria	Case 1	Ampicillin	22 mg/kg IV	TID/Five days	Described as ineffective due to the lack of clinical improvement	Two weeks after treatment/MAT, urine culture, and clinical evaluation.
				Enrofloxacin	2.5 mg/kg IV	BID/Five days	Described as effective therapy due to clinical improvement	
				Amoxicillin/potassium clavulanate	18 mg/kg PO	BID/Four weeks		
				Enrofloxacin	2.1 mg/kg PO	BID/Two weeks	Additional prescription	
				Doxycycline	7 mg/kg PO	Four weeks		
			Case 2	Amoxicillin	12.5 mg/kg PO	BID/Three weeks	Described as effective therapy due to clinical improvement	70 days after treatment/MAT and Clinical evaluation. **Leptospiral shedding not evaluated.**
				Doxycycline	4.6 mg/kg PO	BID/Ten weeks	Additional prescription	
[51]	Cat	Hematuria	1	Amoxicillin	20 mg/kg PO	BID/12 days	Described as effective therapy due to clinical improvement. **Interruption of leptospiruria.**	12, 26 and 60 days after treatment/Urine qPCR
				Doxycycline	10 mg/kg PO	Two weeks		
[52]	Dog	Polyuria and polydipsia	Case 1	Doxycycline	9.2 mg/kg PO	One month	Described as ineffective due to persistence of azotemia and leptospiruria.	28 days after treatment/Urine PCR
				Enrofloxacin	8 mg/kg PO	BID/Three weeks	Described as effective therapy due to clinical improvement. **Interruption of leptospiruria.**	Seven days after treatment/Urine PCR and clinical evaluation
		Polyuria, polydipsia, intermittent vomiting, and loose stool	Case 2	Ampicillin	25 mg/kg IV	QID/One week	Described as effective due to clinical improvement, but leptospiruria persisted	
				Doxycycline	6.5 mg/kg PO	BID/48 days	Described as ineffective due persistence of leptospiruria	3, 10, 18, and 48 days following discharge/Urine PCR
				Enrofloxacin	6.5 mg/kg PO	BID/Two weeks	Described as effective due **interruption of leptospiruria**	Two weeks after treatment/Urine PCR
		Lethargy, anorexy, mild dehydration, and azotemia.	Case 3	Ampicillin/Sulbactam and Enrofloxacin	22 mg/kg IV (Ampicilin/Sulbactam); 10 mg/kg IV (Enrofloxacin)	QID/Nine days (Ampicilin/Sulbactam); SID/Nine days (Enrofloxacin)	Described as effective due to clinical improvement, but leptospiruria persisted	14, 19, 36, and 69 days after treatment/Urine PCR and clinical evaluation
				Amoxicillin/potassium clavulanate and Enrofloxacin	20 mg/kg PO (Amoxicillin); 4.7 mg/kg PO (Enrofloxacin)	BID/Five days (Amoxicillin); SID/Five days (Enrofloxacin)		
				Doxycycline	10 mg/kg PO	SID/12 days		
				Clarithromycin	11 mg/kg PO	BID/33 days	Described as effective due **interruption of leptospiruria**	
		Fever, non-specific and general malaise	Case 4	Ampicillin/Sulbactam and Enrofloxacin	22 mg/kg IV (Ampicilin/Sulbactam); 10 mg/kg IV (Enrofloxacin)	QID/Seven days (Ampicilin/Sulbactam); SID/Seven days (Enrofloxacin)	Described as ineffective due to persistence of leptospiruria.	Seven and 13 days after treatment/Urine PCR and clinical evaluation
				Clarithromycin	9.6 mg/kg PO	BID/Two weeks	Described as effective due **interruption of leptospiruria**	
		Polyuria and polydipsia	Case 5	Amoxicillin/Clavulanic acid	11.4 mg/kg PO	BID/One week	Described as ineffective to improve clinical conditions and interrupt leptospiruria	Seven and 13 days after treatment/Urine PCR and clinical evaluation
				Enrofloxacin	6.2 mg/kg PO	BID/One week	Described as effective due to clinical improvement, but leptospiruria persisted	Seven, ten and 22 days after treatment/Urine PCR and clinical evaluation
				Clarithromycin	10.9 mg/kg PO	BID/Three weeks	Described as effective due **interruption of leptospiruria**	
[26]	Dog	Fever, joint or muscle pain, anorexia, vomiting and diarrhea, nasal and ocular discharge, polyuria and jaundice	45	Enrofloxacin	10 mg/kg IM	SID/Ten days	Described as effective due to clinical improvement and interruption of leptospiruria	30 days after treatment/Urine PCR
			45		10 mg/kg PO	SID/One week		
[53]	Dog	Fever, diarrhea with hematochezia, anorexia, vomiting and dehydration	1	Ampicillin and Enrofloxacin	25 mg/kg SC (Ampicillin) and 5 mg/kg SC (Enrofloxacin)	Single dose	Described as ineffective to improve clinical conditions	Seven, 32 days afets treatment/Hematological and clinical evaluation. **Leptospiral shedding not evaluated.**
				Ampicillin and Cefotaxime	5 mg/kg IV (Ampicillin) and 50 mg/kg IV (Cefotaxime)	11 days	Described as effective due to clinical improvement and interruption of leptospiruria	

In contrast to the protocols observed in livestock, treatment approaches for dogs and cats included not only monotherapy and combination therapy, but also sequential monotherapy. This approach involves the consecutive use of different antimicrobials, administered without temporal overlap, aiming to achieve clinical cure. Sequential therapy was typically guided by the animal’s clinical response, allowing adjustments to the antimicrobial regimen based on improvement or disease progression. Sequential therapy was employed in both dogs and cats, with the number of drugs ranging from two to six in dogs [52] and from two to five in cats [50]. Among the eight studies reviewed, the majority (5/8; 65.5%) reported the use of sequential therapy. Monotherapy was used in three studies (3/8; 37.5%), while combination therapy was implemented within some sequential regimens, occurring in three studies (3/8; 14.3%). Some studies included multiple clinical cases, in which different treatment strategies were used—for instance, sequential therapy in one case and monotherapy in another. Additionally, certain sequential protocols incorporated combination therapy at one or more stages.

The most frequently used antimicrobial was doxycycline, reported in six out of eight studies (75%), in both dogs and cats. Aminopenicillins (ampicillin or amoxicillin) were the second most common, used in 5/8 studies (62.5%), also across both species. Enrofloxacin ranked third, appearing in 3/8 studies (37.5%) for the treatment of both dogs and cats. Other antimicrobials were used less frequently, including penicillin, amoxicillin combined with clavulanic acid, streptomycin, oxytetracycline, chlortetracycline, clarithromycin, and cefotaxime combined with ampicillin.

#### 3.2.1. Tetracyclines

The tetracyclines used in dogs and cats were doxycycline, oxytetracycline, chlortetracycline, and minocycline.

Doxycycline was administered in four studies involving dogs and in two studies involving cats. In all cases, doxycycline was administered orally, with treatment durations ranging from 14 to 49 days. However, there was no standardized dosage established for either species.

In dogs, the effectiveness of bacteriological treatment varied between studies. Juvet et al. [49] reported that doxycycline improved clinical and biochemical parameters but failed to eliminate leptospiral shedding in urine. Similarly, Mauro and Harkin [52] treated four dogs with doxycycline, but the therapy was later replaced to achieve clinical improvement. Two studies used a dosage of 5 mg/kg for different treatment durations. One of them showed clinical resolution after 21 days [53], while others observed that three dogs eventually tested negative by urine PCR during treatment. Doxycycline was used as a first-line drug in two of the cases reported by Mauro and Harkin [52], but it was changed to another antimicrobial. For cats, Arbour et al. [50] reported clinical improvement using doxycycline at 7 mg/kg for 28 days. Another study demonstrated that a dosage of 10 mg/kg for 14 days resulted in negative PCR results [51].

Oxytetracycline and chlortetracycline were used as monotherapy to treat experimentally infected dogs [48], administered orally at a dosage of 25 mg/lb for five days. Both treatments resulted in 80% clinical improvement in their respective groups; however, urinary shedding was not assessed using a sensitive diagnostic method.

#### 3.2.2. Beta-Lactams

The beta-lactams used in dogs and cats were amoxicillin, ampicillin, penicillin, and the combinations amoxicillin + clavulanic acid, ampicillin + cefotaxime.

Aminopenicillins, specifically ampicillin and amoxicillin, were used as first-line treatments in both dogs [52] and cats [50,51], but were later replaced due to insufficient clinical improvement. Ampicillin was primarily administered intravenously in hospitalized animals, with dosages ranging from 22 to 25 mg/kg in both species. Treatment duration varied from 5 to 11 days. In one study, ampicillin was combined with enrofloxacin in a single subcutaneous dose, but this protocol did not lead to clinical improvement [49].

Amoxicillin was used only in cats and administered orally, with treatment protocols of either 12.5 mg/kg for 21 days or 20 mg/kg for 12 days. Nonetheless, all aminopenicillin-based treatments mentioned above were eventually substituted with other drugs due to inadequate therapeutic response.

Amoxicillin combined with clavulanic acid was used in two studies involving dogs, though dosages and durations were not standardized. Reported dosages ranged from 11.4 to 20 mg/kg, with treatment durations between 7 and 28 days. In both cases, the therapy was ultimately discontinued. Ampicillin combined with enrofloxacin was administered as a single subcutaneous dose in dogs [49], and the same combination was used intravenously in a cat [50], however, neither study reported any clinical improvement.

Penicillin G was administered to experimentally infected dogs at a dosage of 50,000 IU/lb for five days, resulting in clinical improvement in 83.3% of the animals in that group [48]. In another study, penicillin was given intravenously at 10 mg/kg for five days, but no clinical improvement was observed, and the treatment was subsequently replaced [53].

#### 3.2.3. Enrofloxacin

Enrofloxacin is a fluoroquinolone that has been administered to dogs [26,52] and cats [50]. This drug has shown efficacy in resolving clinical signs and achieving negative PCR results in urine samples. In the study by Mauro and Harkin [52], enrofloxacin was administered to two dogs that were unresponsive to other antibiotics. Two effective oral protocols were used: 6 mg/kg for 14 days or 8 mg/kg for 21 days. However, a third dog in the same study received 6.2 mg/kg orally for only 10 days. That dog continued shedding leptospires in urine, suggesting that a shorter treatment duration may be less effective. Gutierrez et al. [26] conducted a clinical trial in which 45 dogs received enrofloxacin at 10 mg/kg intramuscularly for 10 days, followed by the same dose orally for more seven days. After completing the protocol, all treated animals tested negative for leptospiral DNA by PCR.

In cats, enrofloxacin was used as a first-line treatment at a dose of 2.5 mg/kg intravenously for 5 days [50]. However, due to a lack of clinical improvement, the drug was subsequently replaced by another antimicrobial agent.

#### 3.2.4. Clarithromycin

Clarithromycin is a macrolide that was used in only one study [52]. The drug showed effective results in both clinical improvement and the interruption of leptospiral shedding. In this study, the authors administered clarithromycin at 9.6 mg/kg for three weeks in one dog and 10.9 mg/kg for three weeks in another. Both dogs became PCR-negative for leptospiral DNA in urine following treatment. In both cases, clarithromycin was used as a last-resort option after failure of previous antimicrobial therapies in a sequential treatment approach.

### 3.3. Antimicrobial Treatment of Leptospirosis in Horses

A total of five studies were selected, all involving naturally infected equines presenting a range of clinical manifestations. In several studies, animals displayed distinct clinical signs and therefore received different treatment protocols. Four studies focused on the treatment of equines with acute kidney disease and/or hematuria [25,54,55,56], with Bernard et al. [55] also reporting a case involving a mare with abortions and acute myalgia. Another study evaluated therapeutic approaches in mares diagnosed with placentitis [57] (Table 3).

Across these studies, 12 distinct treatment protocols or drug combinations were employed. In some cases, different protocols were applied to individual animals within the same study. Similarly toSimilar to the therapeutic approaches used in cats and dogs, treatments in equines were categorized as monotherapy (two studies), combined therapy (five studies), or sequential therapy (two studies). The drugs included beta-lactams (e.g., penicillin, ticarcillin, cefquinome), aminoglycosides (e.g., streptomycin, amikacin, gentamicin), tetracyclines (e.g., doxycycline, oxytetracycline), as well as enrofloxacin and a combination of sulfamethoxazole and trimethoprim. Of the six studies, only four evaluated whether the therapy interrupted the leptospiral shedding, and not only the clinical improvement.

#### 3.3.1. Beta-Lactams and Associations

Penicillin was used in all six studies, predominantly in combination with other drugs; however, one study administered it as monotherapy in a single animal. In that case, penicillin was given intravenously at a dose of 20,000 IU/kg for seven days. In the remaining studies, penicillin was combined with various antimicrobials, including streptomycin, amikacin, gentamicin, oxytetracycline, doxycycline, sulfamethoxazole-trimethoprim, and enrofloxacin. The penicillin dosage ranged from 10,000 to 200,000 IU/kg, administered either intramuscularly or intravenously.

The combination of penicillin and amikacin (25 mg/kg IV for 14 days) proved effective in halting bacterial shedding and improving clinical signs in a foal with hematuria [55]. Similarly, the association of penicillin and streptomycin (25 mg/kg IM for 7 days) successfully treated mares with placentitis and eliminated bacterial shedding [57]. The association of penicillin with gentamicin (2.2 mg kg) in the intravenous route for four days showed clinical improvement in a horse, in which the association of penicillin and sulfamethoxazole-trimetrexate (20 mg kg orally for 3 days) had previously failed [54]. An association of penicillin and enrofloxacin intravenously was also tested in foals with acute kidney disease and was effective in improving clinical aspects.

Other beta-lactam, ticarcillin, combined with clavulanic acid (40 mg/kg IV for 6 days), was effective in reducing clinical signs and stopping bacterial shedding in a horse with acute kidney disease. Cefquinome was employed as monotherapy either as a first-line treatment or following penicillin failure in equines with acute kidney disease. In one study, cefquinome was administered intravenously at 2 mg/kg for 10 days [25].

#### 3.3.2. Other Drugs

A high-dose streptomycin regimen (50 mg/kg for three days), followed by oxytetracycline (5 mg/kg for five days), was administered to a mare that had aborted. Following treatment, the mare tested negative for leptospiral shedding in urine by FAT [55]. In the same study, two asymptomatic but FAT-positive horses were treated with oxytetracycline alone for five days, and also tested negative after therapy. Oxytetracycline was additionally used intravenously (20 mL of a 200 mg/mL solution) for five days in mares with placentitis [57]. However, among the seven treated mares, two remained FAT-positive in urine samples after treatment.

Two studies prescribed oral antimicrobial therapy upon discharge, even when the animals were clinically healthy at that time, due to the severity of the initial presentation. In one case, streptomycin (10 mg/kg; duration not specified) was recommended for a horse with acute kidney disease [54]. In another, doxycycline (10 mg/kg PO for 21 days) was prescribed as follow-up treatment for horses previously treated for acute kidney disease [25].

## 4. Discussion

Leptospirosis in animals presents a wide spectrum of clinical manifestations. Ruminants and pigs often exhibit chronic or subclinical infections [6,58], while dogs and horses are more prone to acute presentations [16,59]. Although cats can develop systemic signs, these are typically milder compared to those observed in dogs [60]. These interspecies differences significantly influence therapeutic strategies. In livestock, diagnosing and treating asymptomatic individuals is a common practice for controlling infection at the herd level [6]. In contrast, treatment protocols for dogs and horses are generally focused on managing severe, acute cases, often requiring hospitalization and repeated therapeutic interventions [16,59]. These interventions primarily aim to resolve the acute condition, focusing on clinical stabilization, reversal of organic alterations, and prevention of severe complications, particularly renal and hepatic failure. It is important to emphasize the clear distinction in treatment protocols and outcome evaluation between clinically ill animals and asymptomatic carriers—a difference that is not only conceptual but also practical and biologically relevant. In clinical cases, the literature [6] indicates that the choice of antimicrobial, route of administration, and duration of therapy should prioritize the rapid reduction in bacterial load and the resolution of systemic inflammatory responses, thereby decreasing the risk of renal and hepatic failure. These contrasting approaches highlight the need for specific guidelines that take into account host biology and therapeutic objectives.

Since there are different purposes of treatment, the strategic usage of bacteriostatic or bactericidal antimicrobials is particularly relevant when interpreting treatment outcomes in leptospirosis. In acute clinical infections, bactericidal drugs may be advantageous since they promote a rapid reduction in bacterial load and reduce the risk of severe renal and hepatic failure. In contrast, in chronic carriers, where the treatment aims to mitigate transmission, bacteriostatic agents may still play a role by limiting bacterial replication and facilitating host immune clearance. However, the available literature rarely makes this distinction explicit, and comparative studies assessing the clinical impact of antimicrobial mode of action remain scarce. This highlights the need for further investigations directly evaluating whether bactericidal versus bacteriostatic therapies result in different outcomes in acute versus chronic leptospiral infections.

In this study, we found that most of the studies identified in our review were published before 2000, highlighting a significant gap in clinical trials aimed at standardizing leptospirosis treatment over the past 25 years. This may reflect the neglected status of leptospirosis, and the scarcity of comprehensive recent studies reinforces this concern. Notably, all studies involving swine and half of the equine studies included in our review were conducted before 2000. While clinical trials and experimental studies are most recommended for establishing effective therapeutic protocols, only the studies of ruminants, swine, and two studies involving dogs used this approach. The remaining studies focused on the treatment of naturally occurring clinical cases, which are usually associated with successive treatment attempts, making it difficult to determine the specific efficacy of each antimicrobial agent. This represents a major challenge in evaluating and standardizing therapeutic approaches for leptospirosis.

Another important aspect observed herein was the wide variety of antimicrobials and treatment protocols applied across different countries. In the United States, for example, cattle studies have evaluated a broad range of drugs, including streptomycin, penicillin, oxytetracycline, tylosin, tilmicosin, tulathromycin, and ceftiofur. The heterogeneity found herein suggests the absence of a standardized national recommendation. One case example is India, which has issued national guidelines that provide comprehensive recommendations encompassing bovines, swine, equines, dogs, and cats [61,62]. However, the dosage of streptomycin indicated in the Indian guideline (12 mg/kg) [62] does not align with the doses commonly reported in the scientific literature (25 mg/kg). Similarly, New Zealand has adopted national policies for the treatment of bovine semen and embryo donors, recommending long-acting oxytetracycline at 20 mg/kg [63]. In the United Kingdom Commonwealth, export regulations for bovine semen stipulate that bulls must receive two doses of streptomycin at 25 mg/kg, administered 14 days apart [64]. Taken together, these examples highlight differences between official guidelines and published literature, and the lack of harmonization among countries. They underscore the urgent need for standardization of treatment protocols and for the implementation of evidence-based governmental guidelines, particularly in regions where leptospirosis remains highly prevalent.

Regarding treatments by animal species, therapies in ruminants, particularly cattle, have been extensively investigated due to the substantial economic losses caused by leptospirosis [4]. Historically, research efforts have focused primarily on renal carriers, leading to the development of therapeutic protocols mainly focused on eliminating kidney colonization. However, recent evidence of genital tract colonization in livestock [13,65,66] has shifted the discussion toward the need for updated treatment strategies targeting genital colonization. Consequently, treatment protocols in ruminants have been more frequently reviewed and refined, with streptomycin remaining the most tested antibiotic. This aminoglycoside has demonstrated high efficacy in eliminating both renal colonization [30] and genital colonization in sheep, and cattle [33,47], although in different protocols. Streptomycin has also shown promising outcomes in swine [42]. Its effectiveness is attributed to potent bactericidal activity, the ability to achieve therapeutic concentrations in target tissues, and rapid inhibition of bacterial protein synthesis [20]. Nevertheless, its use is limited by regulatory restrictions and mandatory withdrawal periods in dairy and meat-producing animals. Concerns over antimicrobial residues in food products, especially from streptomycin, have led many countries to restrict or prohibit its use. Streptomycin is currently not licensed in Australia [67] and is subject to severe restrictions in the United States [68], New Zealand [69], and the European Union [70]. These limitations make standardized treatment protocols based on streptomycin unfeasible in such regions.

Oxytetracycline, a bacteriostatic tetracycline, is a more accessible alternative for livestock and is available in multiple formulations and routes of administration [71], including intramuscular, intravenous, intrauterine, topical, and oral (via drinking water or feed). Long-acting injectable formulations have shown promising results in interrupting leptospiral infection [24,35]. However, efficacy depends on both the route and formulation; for example, oral administration via feed has yielded unsatisfactory results in swine with persistent leptospiruria [34]. Given its broader availability worldwide, oxytetracycline warrants further investigation to define standardized, effective treatment regimens for ruminants. Another drug, Ceftiofur, which is a third-generation cephalosporin with bactericidal activity [72], also emerges as a promising candidate for new ruminant treatment protocols. Studies have reported satisfactory outcomes in bovines [18,32], and certain formulations benefit from short withdrawal periods. Its wider availability across countries strengthens its potential as a practical alternative in regions where streptomycin use is restricted.

While treatment protocols for ruminants are comparatively well established, with defined dosages and durations, the same cannot be said for swine, where recent studies and standardized therapeutic guidelines are lacking. Moreover, existing studies on leptospiral treatment in swine have focused exclusively on clearing renal infection [23,35]. However, genital infection has also been reported in sows [73,74] and appears to be relevant in this species, as in ruminants. This highlights the need for new protocols designed not only to eliminate renal colonization but also to address potential genital tract colonization. An additional consideration in swine is the frequent use of oral administration via feed or water, as demonstrated by Doherty and Baynes [34] and Edwards and Daines [41]. This type of administration requires careful standardization of dosage, formulation, and delivery. Post-treatment evaluation is also essential to ensure reproducible and effective outcomes.

Regarding companion animals such as dogs and cats, treatment is often more complex than in livestock species, as it is typically guided by clinical improvement. Therapeutic approaches in these cases frequently involve sequential administration of multiple successive antimicrobials, with some drugs being discontinued due to a lack of clinical response. Doxycycline and aminopenicillins are the most commonly used agents, and their combination is currently recommended in treatment guidelines for these species [16]. However, several case reports have documented therapeutic failures and persistent leptospiruria in animals treated with these antimicrobials [52,53]. In some instances, treatment inefficacy was so severe that euthanasia was required due to the absence of clinical improvement and the associated welfare concerns [26,53,75]. Another promising drug is enrofloxacin. In their clinical trial, Gutierrez and collaborators [26] successfully achieved bacteriological cure in dogs, which reinforces the potential of this drug to treat leptospirosis.

Noteworthy, the protocols of studies analyzed herein that used doxycycline and or aminopenicillin lacked a standardization of dosage, duration, and route of administration. The majority of studies used the oral administration of those antimicrobials, which raises concerns about proper administration by pet owners, as it may compromise treatment efficacy. This is a critical issue that reinforces the need for randomized clinical trials, with controlled doses and administration routes, to validate (or refute) the currently employed protocols. Additionally, gastrointestinal side effects of doxycycline [76] were frequently reported in dogs, which can compromise treatment adherence and raise ethical concerns regarding patient allocation in clinical studies.

In equines, treatment protocols are even more heterogeneous. Although penicillin often serves as the therapeutic backbone, it is commonly combined with aminoglycosides, fluoroquinolones, or tetracyclines, and the clinical efficacy of these combinations varies widely [59]. A major limitation is that most studies have assessed only clinical and hematological improvement, with few evaluating leptospiral shedding after treatment. Similarly to the situation in companion animals, there is a clear lack of controlled clinical trials on leptospiral treatment in equines. Given the broad spectrum of clinical presentations in this species, standardized protocols should ideally be developed for each form of the disease: reproductive, renal, and respiratory. Noteworthy that treatments for uveitis were not included in this review, as these often rely more heavily on non-antimicrobial therapies, and ocular clearance is difficult to evaluate [77]. Finally, withdrawal periods in equines, particularly due to doping regulations, must be taken into account, further reinforcing the urgency of defining effective and safe protocols for this species.

Beyond the cited antimicrobials, recent in vitro studies have evaluated the activity of next-generation antimicrobials against the *Leptospira* genus [78,79]. In this context, compounds such as minocycline, tigecycline, ertapenem, doripenem, and gatifloxacin have demonstrated potential bactericidal activity, which characterizes them as potential drugs to treat leptospirosis [76,80]. However, the use of these drugs should be considered with caution, since the disease is usually highly responsive to first-line antibiotics, such as penicillins and tetracyclines, which remain effective if well-established in clinical and animal production protocols. In addition, the use of those next-generation compounds may increase the selection of resistant strains and should be reserved for the management of serious infections caused by multidrug-resistant bacteria.

Within this scope, antimicrobial resistance is a critical concern, particularly as a consequence of the indiscriminate use of these drugs, which can select resistant bacteria within the host microbiota and facilitate the transfer of resistance genes [81,82]. Regarding antimicrobial resistance in *Leptospira* spp., the current knowledge remains scarce, especially due to the challenges in cultivating this bacterium. Nonetheless, some in vitro studies reported that *Leptospira* spp. may be resistant to fosfomycin, nalidixic acid, rifampicin, and trimethoprim/sulfamethoxazole [83], and present intermediate sensitivity to chloramphenicol [81]. Furthermore, a recent genomic study has identified resistance genes that suggest resistance to aminoglycosides, beta-lactamases, and vancomycin [82]. This same study has demonstrated the genetic identification of several drug efflux pumps, which might play a role in leptospiral resistance [82]. These outcomes reinforce the need for a better understanding of leptospiral resistance patterns, particularly in leptospires recovered from domestic animals.

In summary, it is notable that the absence of standardized protocols may be associated with a historical neglect of animal leptospirosis. This neglect not only perpetuates economic losses and animal suffering but also poses a threat to public health. Therefore, there is an urgent need to develop therapeutic guidelines based on robust evidence, with rigorous assessment of therapeutic success, not only by clinical signs but through documented eradication of the infectious agent.

## 5. Conclusions

This scoping review highlights that antimicrobial treatment of leptospirosis in domestic animals is characterized by significant heterogeneity, with no standardized protocols established for most species. By compiling and analyzing scattered data across species and regions, our study contributes beyond a descriptive scope: it identifies converging practices that may represent consensus, highlights discrepancies that reveal gaps in knowledge, and provides an evidence-based foundation to guide future harmonization of treatment protocols. The scarcity of randomized clinical trials, combined with insufficient or inadequate post-treatment monitoring, particularly regarding the clearance of bacterial shedding, has limited accurate assessments of treatment efficacy. Thus, this review contributes not only by mapping current practices but also by establishing an evidence-based framework to guide the development of standardized, species-specific therapeutic guidelines, which are urgently needed in a One Health and economic context.

## Figures and Tables

**Table 3:** Description of studies and therapeutic protocols for leptospirosis in equines.

Reference	Species	Clinical	N	Antimicrobial	Dosage	Duration of Treatment	Therapeutic Outcome	Revaluation (Period/Test)
[54]	Equine	Acute kidney disease (Natural infection)	1	Trimethoprim/sulfadiazine and penicillin	Trimethoprim/sulfadiazine (20 mg/kg) VO and Penicilin (18,000 UI/kg) IM	BID/three days	Described as ineffective due to the lack of clinical improval	Three days/Clinical evaluation. **Leptospiral shedding not evaluated.**
					Penicilin (20,000 UI/kg) and Gentamicin (2.2 mg/kg) IV	Penicilin QID/Four days and Gentamicin TID/Four days	Described as effective therapy due to clinical improval	Six months after treatment/Clinical evaluation and MAT. **Leptospiral shedding not evaluated.**
[55]	Equine	Abortion, fever, myalgia and limb claudication (Natural infection)	3	Streptomycin and Oxytetracycline	Streptomycin (50 mg/kg) and Oxytetracycline (5 mg/kg) IM	Streptomycin three doses and Oxytetracycline five doses	Described as ineffective in all animals. Persistent leptospiruria, but clinical improvement is also reported.	For 15 weeks/Urine culture and FAT urine, reproductive clinical evaluation
[57]	Equine	Placentitis (Natural infection)	6	Penicillin and streptomycin	Penicilin (200,000 UI) and streptomycin (250 mg/mL) IM	BID/one week	Described as effective therapy in all animals. **Interruption of leptospiruria.**	NA/Urine culture and FAT
			7	Oxytetracycline	200 mg/mL IV	Five doses	Described as effective in 5/7 (71.4%) of animals. **Interruption of leptospiruria.**	One and three weeks after treatment/Urine PCR
[56]	Equine	Fever, leukocytosis, azotemia, kidney enlargement, acute nephritis, and hematuria (Natural infection)	2	Penicillin and enrofloxacin	Penicilin (30,000 UI/kg) and enrofloxacin (6 mg/kg) IV	Penicilin QID/22 days and Enrofloxacin 21 days	Described as effective therapy due to clinical improval	Three months after treatment/MAT and hematological evaluation. **Leptospiral shedding not evaluated.**
		Fever and mildly altered renal biochemistry (Natural infection)	1			Penicilin QID/ten days and Enrofloxacin seven days	Described as effective therapy due to clinical improval	Three months after treatment/MAT and hematological evaluation. **Leptospiral shedding not evaluated.**
[25]	Equine	Acute kidney disease (Natural infection)	1	Penicillin, followed by doxycycline	Penicilin (20,000 UI/kg) IV and doxycycline (10 mg/kg) VO	Penicilin QID/one week and doxycycline BID/three weeks	Described as effective therapy due to clinical improval	NA/Clinical evaluation. **Leptospiral shedding not evaluated.**
			1	Cefquinome, followed by doxycycline	Cefquinome (2 mg/kg) IV, and doxycycline (10 mg/kg) VO	Cefquinome BID/ten days and	Described as effective therapy due to clinical improval	NA/Clinical evaluation. **Leptospiral shedding not evaluated.**
			1			doxycycline BID/three weeks		
			1	Penicillin (therapeutic failure), Cefquinome, followed by doxycycline	Penicilin (20,000 UI/kg) IV, Cefquinome (2 mg/kg) IV and doxycycline (10 mg/kg) VO	Penicilin QID/six days, Cefquinome BID/one week and doxycycline BID/three weeks	Described as effective therapy due to clinical improval	NA/Clinical evaluation. **Leptospiral shedding not evaluated.**

## Data Availability

Not applicable.
